# Nondestructive nanofabrication on Si(100) surface by tribochemistry-induced selective etching

**DOI:** 10.1038/srep16472

**Published:** 2015-11-12

**Authors:** Jian Guo, Bingjun Yu, Lei Chen, Linmao Qian

**Affiliations:** 1 Tribology Research Institute, Key Laboratory of Advanced Technologies of Materials (Ministry of Education), Southwest Jiaotong University, Chengdu 610031, Sichuan Province, P. R. China

## Abstract

A tribochemistry-induced selective etching approach is proposed for the first time to produce silicon nanostructures without lattice damage. With a ~1 nm thick SiO_x_ film as etching mask grown on Si(100) surface (Si(100)/SiO_x_) by wet-oxidation technique, nano-trenches can be produced through the removal of local SiO_x_ mask by a SiO_2_ tip in humid air and the post-etching of the exposed Si in potassium hydroxide (KOH) solution. The material removal of SiO_x_ mask and Si under low load is dominated by the tribochemical reaction at the interface between SiO_2_ tip and Si/SiO_x_ sample, where the contact pressure is much lower than the critical pressure for initial yield of Si. High resolution transmission electron microscope (HRTEM) observation indicates that neither the material removal induced by tribochemical reaction nor the wet etching in KOH solution leads to lattice damage of the fabricated nanostructures. The proposed approach points out a new route in nondestructive nanofabrication.

Due to its excellent mechanical and physical properties, monocrystalline silicon has become the most important structural material for fabricating various nanostructures in photovoltaic devices^1^,^2^, micro/nanoelectromechanical systems^3^,^4^, etc. As the way to realize such nanostructures, the traditional Si-based nanofabrication approaches are faced with lots of technical challenges in resolution, destruction, flexibility, etc. Photolithography is a typical nanofabrication approach with high-throughput in mass production, but its fabrication process is costly and tends to be limited in resolution^5^. In addition, photolithography is not suitable for flexible fabrication of micro-mold and prototype fabrication of microsystems^6^. Nanoimprint lithography is an effective approach for patterning nanostructures with high resolution^7^. However, the structural damage and stamping defects easily take place during both the imprinting and demoulding processes. Other reported Si-based fabrication techniques such as focused ion beam (FIB) assisted nanolithography^8^,^9^ and scanning probe microscopy (SPM)-based mechanical scratching or cutting^10^ have the straightforward processes, high resolution and flexibility, but the fabrication-induced damage in the subsurface, such as plastic deformation and amorphization, cannot be avoided^11^,^12^.

Recently, maskless friction-induced nanofabrication has attracted a lot of attention by virtue of its simplicity, flexibility and low-destruction^13^,^14^,^15^,^16^,^17^. In the friction-induced nanofabrication process of silicon, the silicon substrate is partly transformed to amorphous silicon by diamond tip scratching firstly^13^,^14^,^15^,^16^,^17^. Then the amorphous silicon layer can be directly served as an etching mask in potassium hydroxide (KOH) solution to fabricate protrusive nanostructures^14^,^15^,^16^,^17^, or selectively dissolved by hydrofluoric acid (HF) solution to fabricate nano-trenches^17^. However, the structural damage of the nanostructures fabricated by using the friction-induced nanofabrication methods still exists. Since it can degrade the mechanical and physical properties of the nanostructures^18^,^19^, the structural damage is detrimental to various applications. Therefore, how to eliminate or avoid the fabrication-induced nanostructure destruction becomes a significant concern. In addition, nanostructures such as nano-hole and nano-trench can also be realized on the surface of semiconductor material through removing local mask layers with mechanical scratch and subsequent wet etching^20^,^21^,^22^. These findings open a door to develop new SPM-based nanolithography method for fabricating nanostructures with lower fabrication destruction.

In this paper, we report a simple and feasible nondestructive nanofabrication approach on monocrystalline silicon through tribochemistry-induced selective etching. With a ~1 nm thick SiO_x_ film as etching mask grown on Si/SiO_x_ surface by wet-oxidation technique, nano-trenches with required depths can be produced through the removal of local SiO_x_ mask by a SiO_2_ tip in humid air and the post-etching of the exposed Si in KOH solution. Experimental results show that both the ambient humidity and normal load of tip scanning during the fabrication process has remarkable effect on the fabrication depth. Different from the traditional material removal mechanism of the SPM-based mechanical scratching or cutting methods with a diamond tip, the material removal of Si/SiO_x_ under low contact pressure (far from enough to result in the initial yield of silicon) in the present method is determined by the tribochemical reaction at the interface between SiO_2_ tip and Si/SiO_x_ sample. High resolution transmission electron microscope (HRTEM) observation indicates that in the present approach, neither the material removal induced by tribochemical reaction nor the wet etching in KOH solution leads to lattice damage beneath the fabrication area. The nondestructive fabrication capability of this approach is demonstrated by a series of nanostructures on Si(100) surface, such as array of nano-trenches, nanochannels and multilayered nanostructure.

## Results

### Fabrication process of the tribochemistry-induced selective etching

**Figure 1:**
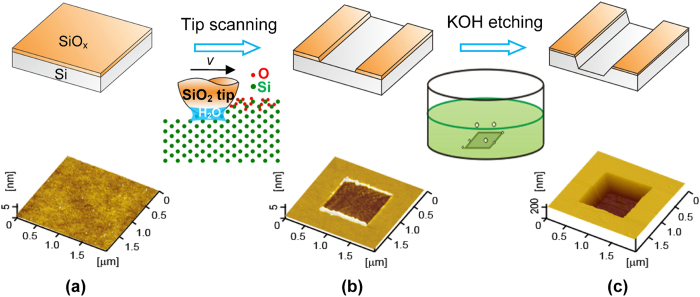
Schematic diagram (top) and fabrication example characterized by AFM (bottom) showing the nondestructive nanofabrication process of tribochemistry-induced selective etching. (**a**) Growing of SiO_x_ film with ~1 nm in thickness on H-passivated Si(100) surface by wet-oxidation technique. (**b**) Scanning a SiO_2_ tip on a Si/SiO_x_ sample in humid air to remove the target SiO_x_ mask. (**c**) Post-etching of the exposed Si in KOH solution to fabricate deep nano-trench.

Figure 1 shows the fabrication process of the tribochemistry-induced selective etching on Si(100) surface. Firstly, SiO_x_ film with thickness of ~1 nm was generated on the H-passivated Si(100) surface through the wet-oxidation technique with hot HNO_3_ solution (Fig. 1a)^23^. Secondly, the local SiO_x_ film on the target area of Si(100) substrate was removed through the scanning of SiO_2_ tip under low load in humid air to expose the Si(100) substrate (Fig. 1b). It has been clarified that under the mechanical shear by the SiO_2_ tip in humid air, the tribochemical wear occurs^24^,^25^,^26^. During the wear process, Si-O-Si bridges firstly form at the contact interface, then water molecules hydrolyze and dissociate the strained Si-O-Si bonding bridges easily to produce the silane compounds that can be removed by tip scanning. As a result, both the SiO_x_ layer and silicon substrate on the scanned area can be gradually removed by such tribochemical reaction. Thirdly, the exposed Si was selectively etched by KOH solution to form a deep nano-trench (Fig. 1c). Due to its lower etching rate in KOH solution than Si(100), the SiO_x_ on the non-scanned area was served as a mask to protect the Si(100) substrate below from etching. Under the present experimental condition, the etching selectivity of SiO_x_:Si(100) was estimated as about 1:250, which was close to the reported selectivity of SiO_2_:Si(100) in KOH solution (less than 1:185^27^).

### Effect of humidity and normal load on the fabrication depth

Experimental results suggested that the fabrication depth depended on not only the KOH etching period but also the relative humidity (RH) and the normal load *F*_n_ during tip scanning process. The *in-situ* three-dimensional topographies of the fabrication areas under various humidity and experiment of load conditions were characterized by atomic force microscope (AFM, Figure S1 and Figure S2 in Supplementary Section 1).

**Figure 2:**
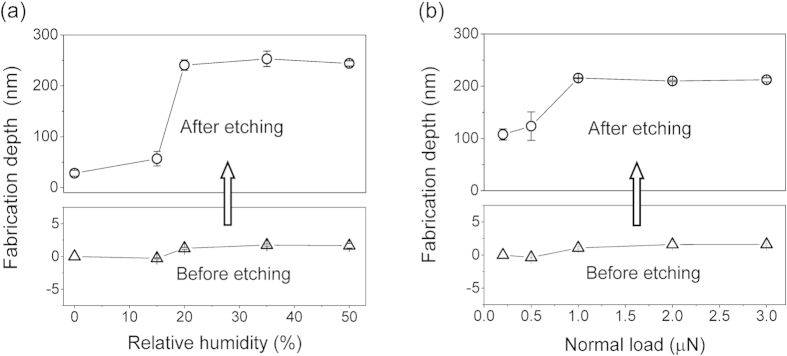
Effect of humidity and normal load on the fabrication depth. (**a**) Fabrication depth vs relative humidity (area-scanning under *F*_n_ of 3 μN, post-etching in KOH solution for 15 min). (**b**) Fabrication depth vs normal load (area-scanning at RH of 50%, post-etching in KOH solution for 10 min).

The fabrication depth (before and after post-etching) versus RH curves are plotted in Fig. 2a. It indicated that under the given load of 3 μN, (i) when RH was 0%, in the absence of tribochemical reaction, no appreciable wear could be observed on the scanned area of SiO_2_ tip from the AFM image. However, because of the slight disturbance of the SiO_x_ structure by mechanical interaction, the disturbed SiO_x_ could be selectively dissolved in KOH solution slowly to obtain a shallow nano-trench; (ii) The tribochemical reaction would be intensified with the increase of RH in certain range (0%–50%), resulting in the increasing material removal (tribochemical wear) of Si/SiO_x_; (iii) There was a threshold RH (~20%) above which the SiO_x_ on the scanned area could be removed thoroughly to expose the Si substrate. Then the exposed Si could be etched by KOH solution to fabricate deeper nano-trench. It was revealed that the tribochemistry-induced nanofabrication could be realized at common humidity condition, without the need for a higher humidity. Figure 2b shows the load dependence of the fabrication depth. The result suggested that load could intensify the tribochemical wear of Si/SiO_x_, and at present experimental condition, a condition of *F*_n_ ≈ 1 μN and one scanning cycle was enough to realize the removal of SiO_x_ mask with ~1 nm in thickness. Once the Si substrate on tip scanning area was exposed, deeper nano-trenches could be produced quickly through the selective etching of Si in KOH solution.

Since the maximum Hertzian contact pressure during the scanning process by SiO_2_ tip (calculated as about 0.48–1.2 GPa) was far below the critical pressure for initial yield of Si (~11.3 GPa^26^), such contact between SiO_2_ tip and Si substrate should be elastic. Moreover, the anisotropic etching of Si in KOH solution is pure chemical behavior^28^, which does not bring lattice distortion beneath the etched nano-trench. Therefore, the subsurface of the fabricated trench was deduced to be composed of damage-free monocrystalline silicon.

### HRTEM characterization on the tribochemistry-induced nanofabrication area

**Figure 3:**
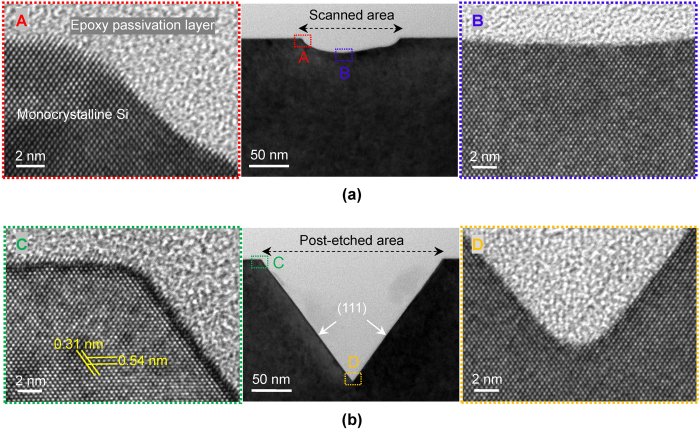
XTEM observation of the tribochemistry-induced nanofabrication area. (**a**) HRTEM image of the scanned area by SiO_2_ tip. (**b**) HRTEM image of the post-etched area by KOH solution. When using epoxy as the passivation layer for preparing the XTEM samples, because the SiO_x_ film (amorphous oxide layer) on silicon substrate surface showed a featureless microstructure similar to that of the epoxy passivation layer, the ~1 nm thick SiO_x_ film on the top surface of silicon substrate was difficult to be identified in TEM images.

In order to verify whether the tribochemistry-induced selective etching nanofabrication brings destruction or not, both the tip scanning area and KOH post-etching area were observed by cross-sectional transmission electron microscope (XTEM), as shown in Fig. 3.

Figure 3a shows the XTEM observation of the scanned area with the SiO_x_ mask removed thoroughly. The observed nano-trench with depth of 18 nm and width of 118 nm (XTEM image, in the middle of Fig. 3a, the cross-section is (110) crystal face of Si) was fabricated on a Si(100)/SiO_x_ sample by line-scanning mode under the conditions of *F*_n_ = 2 μN, number of scanning cycles *N* = 500 and RH = 50%. After the fabrication, the SiO_x_ film and partial silicon substrate beneath the SiO_x_ film on the scanned area were removed because of the tribochemical wear mentioned before. As shown in the HRTEM lattice fringe images, the structure of the scanned area is monocrystalline silicon, no amorphous silicon layer and lattice distortion layer are observed. Figure 3b shows the XTEM observation of the post-etched area by KOH solution. Firstly, a shallow nano-trench was fabricated by line-scanning mode with a SiO_x_ tip under the same fabrication conditions as those of the nano-trench shown in Fig. 3a. Secondly, this sample was dipped into KOH solution etching for 10 min to obtain the final nano-trench with depth of 147 nm and width of 220 nm (XTEM image, in the middle of Fig. 3b). The slope sidewall of nano-trench is (111) crystal face of Si, which is well known to be the etch-stop crystal face due to its ultralow etching rate in KOH solution^28^. It can be clearly observed that the post-etched area is also damage-free monocrystalline silicon without dislocation at all. The result of HRTEM observation of the fabricated area has effectively demonstrated that neither the material removal dominated by tribochemical reaction nor the wet etching in KOH solution results in lattice damage beneath the fabricated area. Such fabricated nanostructure can keep its original single crystal lattice.

### Nondestructive nanostructures fabricated on Si(100)

**Figure 4:**
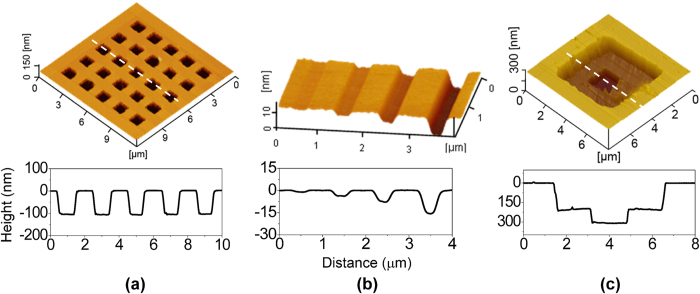
Nondestructive nanostructures on Si(100) surface fabricated by tribochemistry-induced nanofabrication method. (**a**) A 5 × 5 array of nano-trenches. (**b**) Nanochannels. (**c**) Multilayered nanostructure.

Based on the tribochemistry-induced nanofabrication method, a series of nondestructive nanostructures were fabricated on Si(100) surface. Figure 4a shows a high-density 5 × 5 nano-trench array with depth of ~100 nm, which was fabricated by area-scanning under the condition of RH = 50%, *F*_n_ = 3 μN and *t* (post-etching period of KOH solution) = 5 min. Figure 4b shows four nanochannels with the same length of 8 μm and width of 0.5 μm, different depths of about 0.7, 3.8, 7.8 and 15 nm. Such nanochannels were directly produced by area-scanning of SiO_2_ tip under RH = 50%, *F*_n_ = 1 μN and the changed *N* of 1, 2, 4 and 8, respectively. Figure 4c shows a multilayered nanostructure produced on Si(100) surface by carrying out twice tribochemistry-induced selective etching fabrication. Firstly, the first nano-trench with depth of ~190 nm was produced on a Si(100)/SiO_x_ sample under *F*_n_ = 3 μN and *t* = 10 min in the 1^st^ fabrication period. Secondly, after new SiO_x_ mask was grown on the trench area, the second nano-trench with ~130 nm in depth within the first nano-trench was generated under *F*_n_ = 3 μN and *t* = 5 min in the 2^nd^ fabrication period. This fabrication case suggested that nondestructive refabrication on the fabricated surface could be realized through the repetition of tribochemistry-induced nanofabrication process, by which the complex multilevel nanostructures could be fabricated easily.

The fabrication process of tribochemistry-induced selective etching is conductivity-independent and straightforward. Without the need for any additional field (such as electrical field) and template, nano-trenches with arbitrary shape can be precisely patterned on the specified location through programming the trace of tip. By virtue of the near atomic scale tribochemistry wear, the minimum fabrication depth can be below 1 nm (several atoms layer). Different from the traditional SPM-based nanolithography methods for monocrystalline silicon, where the material removal is mainly realized through mechanically scratching or cutting under high load, the tribochemistry-induced material removal process is dominated by tribochemical reaction under much lower load and then does not lead to the lattice damage beneath the fabrication area. A contrasting example of the two different material removal behaviors is shown in the Supplementary Information (Supplementary Section 2). The tribochemistry-induced approach can be used for fabricating nondestructive silicon nano-textures in order to improve or regulate the surface hydrophobicity or tribology properties^29^,^30^,^31^. In addition, the nanochannels free of lattice damage are expected to be applied as the key components in micro/nano fluidic systems^32^, which are widely used in drug delivery, ion transporters, DNA translocators, and so on^33^,^34^,^35^,^36^. The nondestructive tribochemistry-induced nanofabrication method is also available for other chemical reactive surface, such as GaAs, to produce defect-free or well-ordered nanostructures^37^.

## Conclusion

In summary, we have proposed a simple and feasible nanofabrication approach to produce damage-free monocrystalline silicon nanostructures. When the contact pressure does not result in the initial yield of Si, the material removal of Si/SiO_x_ is determined by the tribochemical reaction between the SiO_2_ tip and Si/SiO_x_ sample. Experimental results suggest that the fabrication depth is mainly dependent on the humidity, the normal load during tip scanning process and the etching period in KOH solution. Results of HRTEM observation reveal that the nanostructures fabricated by tribochemistry-induced selective etching are free of lattice damage. Such nondestructive nanostructures are very difficult to be fabricated by conventional SPM-based mechanical cutting or friction-induced nanofabrication methods.

## Experimental

### Materials and methods

Experiments were performed on p-type Si(100) wafers with thickness of 0.5 mm (MEMC Electronic Materials, Inc., USA). Before the fabrication, samples were dipped into 10 wt.% HF solution for 2 min to remove the superficial native oxide layer, and then the Si surface became H-passivated^38^. Because of its strong oxidizing and low viscosity, HNO_3_ solution was used to produce SiO_x_ mask on Si substrate^23^. The H-passivated Si samples were soaked in ~65 wt.% HNO_3_ solution for 30 min at 80 °C, then SiO_x_ film with ~1 nm in thickness (characterized by scanning Auger nanoprobe (PHI 700, ULVAC-PHI, Inc., Japan), see Figure S5 in Supplementary Section 3) was generated on Si(100) substrate. With an AFM (SPI3800N, Seiko, Japan), the root-mean-square roughness of the Si/SiO_x_ sample was measured to be less than 0.08 nm over an area of 500 nm × 500 nm. Spherical SiO_2_ tips (Novascan Technologies, USA) with radii of ~1.0 μm were employed for the scanning to remove the local SiO_x_ mask. The normal spring constant of the cantilever was 14 N/m. In post-etching procedure, a mixture of 20 wt.% KOH solution and isopropyl alcohol (volume ratio = 5:1) was used as an etchant for selective etching of the exposed Si. The etching temperature was set to be 23 ± 2 °C.

### AFM and XTEM characterization

All of the AFM images were scanned by silicon nitride probes (MLCT, Bruker Corp., USA) with a spring constant of 0.1 N/m. The microscopic structural feature of the fabricated area on silicon sample was detected by XTEM (Tecnai G2 F20, FEI, USA). The XTEM samples of the fabricated area were prepared using a FIB (NanoLab 400, FEI, USA) miller. Before FIB cutting, an epoxy polymer passivation layer was deposited on the sample surface to protect the surface from damage in subsequent FIB cutting process.

## Additional Information

**How to cite this article**: Guo, J. *et al.* Nondestructive nanofabrication on Si(100) surface by tribochemistry-induced selective etching. *Sci. Rep.* **5**, 16472; doi: 10.1038/srep16472 (2015).

## Supplementary Material

Supplementary Information
