# Supplementary material for: Nondestructive nanofabrication on Si(100) surface by tribochemistry-induced selective etching
Source: Sci Rep. 2015 Nov 12;5:16472. doi: 10.1038/srep16472 (PMC4642297; doi:10.1038/srep16472)
Supplement: Supplementary Information [file srep16472-s1.pdf]

Supplementary Information

Nondestructive nanofabrication on Si(100) surface by tribochemistry-induced selective etching

Jian Guo, Bingjun Yu, Lei Chen, Linmao Qian*

Tribology Research Institute, Key Laboratory of Advanced Technologies of Materials (Ministry of Education), Southwest Jiaotong University, Chengdu 610031, Sichuan Province, P. R. China

* Corresponding Author: linmao@swjtu.edu.cn, Tel.: +86 28 87600687 and Fax: +86 28 87603142

1. AFM images of the tribochemistry-induced fabrication areas before and after selective etching under various humidity and load conditions

Figure S1a show the AFM images of the fabrication areas by scanning a spherical SiO_2 tip ($R \approx 1$ μm) on a Si(100)/SiO_x sample surface under a normal load of 3 μN at various relative humidity (RHs). Since the thickness of the SiO_x film was about 1 nm, the SiO_x film on the fabrication areas at RHs above 20% was removed and the Si(100) substrate on these areas was exposed. After KOH solution etching for 15 min, above SiO_2 tip fabrication areas were all transformed to deeper nano-trenches, as shown in Figure S1b. The corresponding fabrication depths were plotted in Figure 2a in main text.

Figure S2a shows the AFM images of the fabrication areas by scanning the tip at RH = 50% and under various normal loads. The AFM images of all the fabrication areas after post-etching in KOH solution for 10 min are shown in Figure S2b. The corresponding fabrication depths were plotted in Figure 2b in main text.

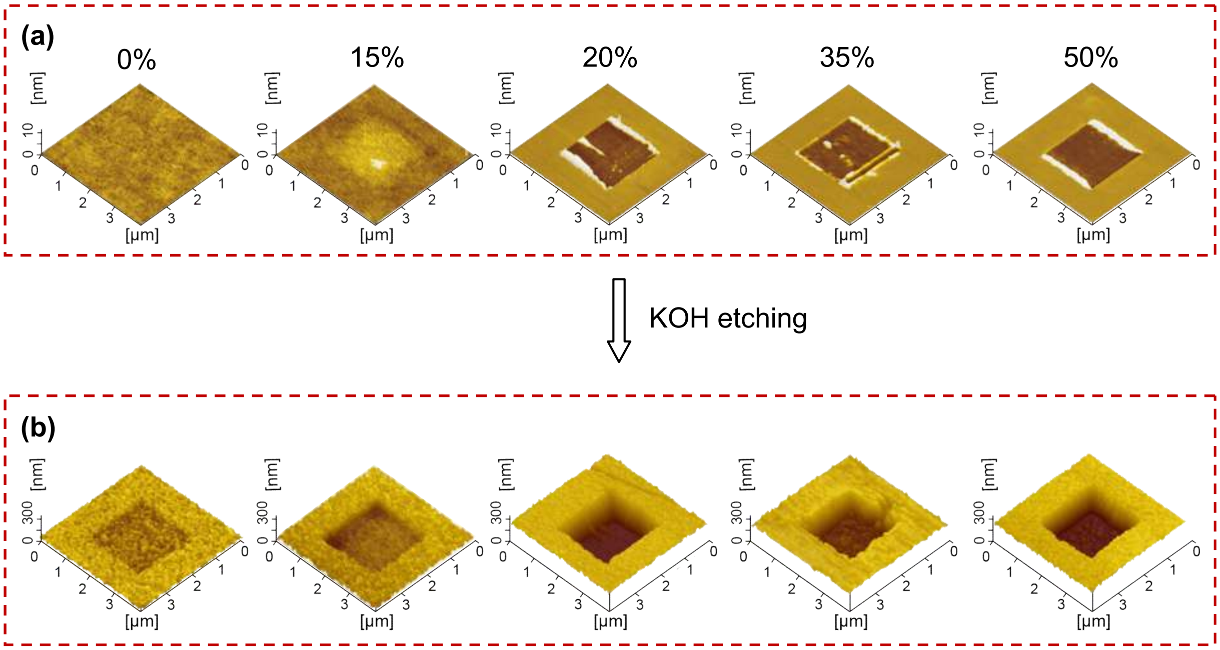

Figure S1. (a) AFM images of scanned areas by SiO_2 tip under a normal load of 3 μN and at various RHs. (b) AFM images of the above scanned areas after post-etching in KOH solution for 15 min.

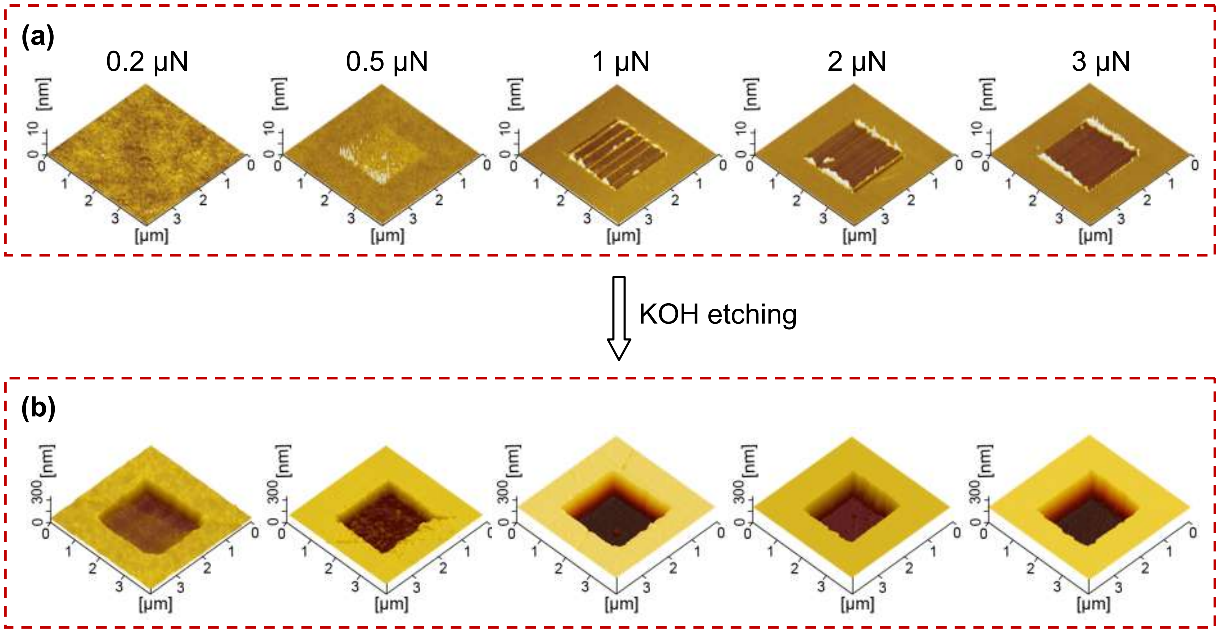

Figure S2. (a) AFM images of scanned areas by SiO_2 tip at RH = 50% and under various normal loads. (b) AFM images of the above scanned areas after post-etching in KOH solution for 10 min.

2. Comparison of the tribochemistry-induced material removal with the traditional SPM-based mechanical cutting

Generally, nano-trenches can be produced on silicon sample surface by sliding a diamond tip or diamond like carbon-coated Si tip along the designed trace, as long as the contact pressure is high enough to result in the yield of silicon.[1-3] Therefore, the fabrication-induced damage in the subsurface cannot be avoided. Different from the mechanical cutting of the diamond tip, the tribochemistry-induced material removal with the SiO_2 tip can be realized when the contact pressure is lower than the critical contact pressure for initial yield of silicon.[4]

Figure S3a shows a nano-trench on Si(100) surface with a depth of ~21.2 nm, produced by a spherical SiO_2 tip with radius R ≈ 1 μm, under the condition of RH ≈ 50%, normal load F_n = 2 μN and number of scanning cycles N = 500. The corresponding Hertzian contact pressure was calculated as ~1 GPa, under which the plastic yield of silicon would not occur and the forming of nano-trench (material removal of Si/SiO_x) was determined by the tribochemical reaction at the interface of SiO_2 tip and Si/SiO_x sample. XTEM observation of this nano-trench area is shown in Figure S4a, which indicates that no amorphous silicon layer and distorted layer can be observed beneath the nano-trench.

Figure S3b shows an irregular nano-trench on Si(100) surface with an averaged depth of 5 nm, fabricated by a spherical diamond tip with R ≈ 5 μm, under the condition of RH ≈ 50%, F_n = 12 mN and N = 1. Since the nominal maximum Hertzian contact pressure (~12 GPa) is higher than the critical contact pressure for initial yield of silicon (~11.3 GPa), the yield of silicon occurs. The corresponding XTEM observation of this area (Figure S4b) shows that a ~230 nm thick amorphous silicon layer and a bottom distorted layer (e.g. dislocation) in a sequence are formed in the subsurface of the nano-trench area (due to its low aspect ratio, the nano-trench is not clear in the XTEM image). Above contrast experimental results reveal that the tribochemical reaction induced by SiO_2 tip is the prerequisite to realize the fabrication of damage-free nanostructures, which are very difficult or impossible to be realized by the traditional mechanical scratching or cutting methods.

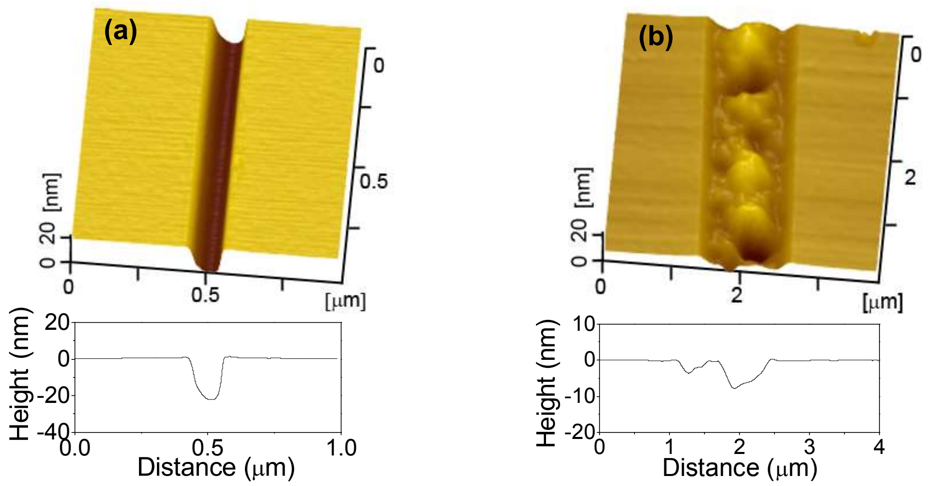

Figure S3. (a) Tribochemistry-induced material removal on Si/SiO_x sample by SiO_2 tip. (b) Mechanical cutting on Si/SiO_x sample by diamond tip. The surface topographies of these fabrication areas were characterized by AFM.

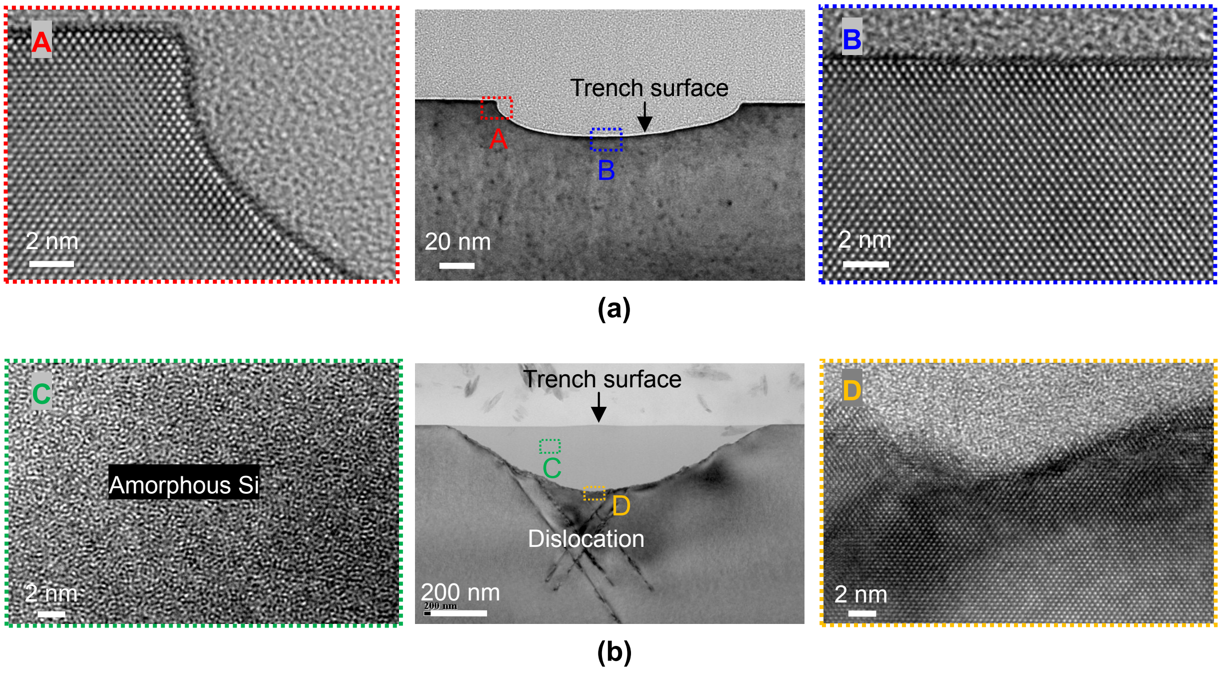

Figure S4. (a) XTEM observation on the nano-trench area (shown in Figure S3a) produced by tribochemistry-induced fabrication on Si(100) surface. (b) XTEM observation on the nano-trench area (shown in Figure S3b) produced by the traditional mechanical cutting fabrication on Si(100) surface.

3. Characterization on the thickness of the wet-oxidation SiO_x mask by scanning Auger nanoprobe analysis

In order to measure the thickness of the wet-oxidation SiO_x mask on Si(100) sample surface, scanning Auger nanoprobe analysis was employed. The atomic concentration and depth distribution of Si and O are shown in Figure S5. It was found that the variation of the atomic concentration of O and Si began to level off (the concentration of O was close to zero) when the depth was about 1 nm. Therefore, we estimated that the thickness of the SiO_x mask was ~1 nm.

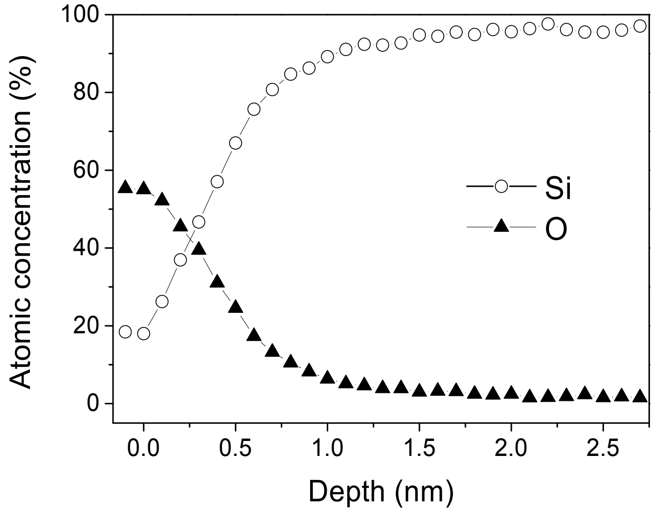

Figure S5. Scanning Auger nanoprobe analysis on the Si/SiO_x sample surface to measure the thickness of the wet-oxidation SiO_x mask.

References

1. Ogino, T., Nishimura, S. & Shirakashi, J. -i. Scratch nanolithography on Si surface using scanning probe microscopy: influence of scanning parameters on groove size. *Jpn. J. Appl. Phys.* **47**, 712 (2008).
2. Ogino, T., Nishimura, S. & Shirakashi, J -i. Nanoscale patterning of Si surface using SPM scratching. *Proceedings of the 17th International Vacuum Congress/13th International Conference on Surface Science/International Conference on Nanoscience and Technology* **100** (2008).
3. Jiang, X. *et al.* Nanopatterning on silicon surface using atomic force microscopy with diamond-like carbon (DLC)-coated Si probe. *Nanoscale Res. Lett.* **6** (2011).
4. Yu, J. X., Kim, S. H., Yu, B. J., Qian, L. M. & Zhou, Z. R. Role of Tribochemistry in nanowear of single-crystalline silicon. *ACS Appl. Mater. Interfaces* **4**, 1585-1593 (2012).
